# Therapeutic Notes

**Published:** 1896-05-16

**Authors:** 


### THERAPEUTIC NOTES.

### LOCAL APPLICATION OF GUAIACOL AS A MEANS OF REDUCING TEMPERATURE.

E. A. Bourke, in the *Medical Press* of April 22nd, 1896, gives an account of the effect of guaiacol when used locally with the object of reducing temperature. The axilla was usually the seat employed for application; it was first washed with soap and tepid water and then dried. The guaiacol was then gently rubbed in with the finger and covered with oil silk to prevent evaporation. In special cases the part chosen may for various reasons be varied; for instance, in typhoid the abdomen seems the best suited, and in phthisis the areas immediately beneath the clavicle over the affected part. The guaiacol may be applied either in solution, or mixed with oil or glycerine; the local treatment is generally followed by slight irritation of the skin, but the effect is only transitory. In enteric fever only a reduction of from one to four degrees of temperature was noticed in a series of eight cases.

In puerperal fever a fall of 2·5 degrees was observed; in fact, a uniformly good result was obtained in every case except in one of phthisis, in which the evening temperature was only about one degree above normal. A peculiar advantage is claimed for this method, in that there appears no tendency for the temperature to mount subsequently, as is usually observed after the usual methods for reducing temperature, such as the application of ice, sponging, and the use of internal febrifuges. Other advantages claimed are that in phthisis the cough is checked, and the sweating is controlled; in typhoid, the quantity of urine passed is increased. In no cases were bad effects noticed such as to contra-indicate this method, possibly because only small quantities were employed; in fact, seldom more than ten minims, and usually between five and ten minims for adults, and from three to five minims for children. The reduction in temperature is generally observable from half to one hour after treatment. In this series the application was confined to the evening, and then only once applied; it is possible that even better results might have been obtained had larger quantities at shorter intervals been employed. Heart failure as the result of over-large doses of the drug is the only danger to be guarded against, but no indication of such a result was noticed in any of the cases above quoted.

### EUROPHEN IN THE TREATMENT OF LEPROSY.

In the *Bulletin Medical*, December 18th, 1895, there is reported a case of leprosy treated by Dr. Goldschmidt, and cured by the use of europhen. The patient had leprous patches on the face and limbs, which were treated locally by the application of pure europhen and olive oil in the proportion of 1 to 19. The oil was rubbed into the patches by brisk friction for periods of ten minutes twice a day, and the treatment was continued for four years. It was also applied on cotton wool at night, with tampons for the nostrils. From an early date the patches showed signs of healing, and the general appearance of the skin appeared more healthy, and this initial improvement encouraged Dr. Goldschmidt to persevere with the method. At the end of four years' continuous treatment he reports that not a trace of the leprosy can be detected. This case has special interest, since it is an actual case of cure. It is the first on record. Europhen is chemically iso-butyl-ortho-cresyl-iodide; it is a yellow amorphous powder, containing 28 per cent. of iodine. It has a slight saffron odour. It has frequently been used with good effect as a dusting powder or ointment in the treatment of simple or venereal sores, and may be used in all cases in which iodoform is indicated. In former contributions Dr. Goldschmidt has given us his experience in the use of europhen in one or two other cases of leprosy, but, perhaps from the fact that they were too advanced, little benefit seems to have accrued from the treatment. The tubercular forms seem to hold out most hopes of successful treatment, since the disease in these cases often remains localised and without general infection for a considerable number of years.

